# 6.G. Workshop: The European Health Data Space 2: is Europe ready to maximise the re-use of health data?

**DOI:** 10.1093/eurpub/ckac129.365

**Published:** 2022-10-25

**Authors:** Organised by: Sciensano (Belgium), Belgian Federal Public Health Institute Towards the European Health Data Space, PHIRI, Chair persons: Irini Kesisoglou (Belgium)

## Abstract

The EU's Heads of State called in October 2020 to set up a European Health Data Space (EHDS): “The European Council welcomes the creation of common European data spaces in strategic sectors, and in particular invites the Commission to give priority to the health data space”. As a response, multiple initiatives were set-up to engage the views and expectations from EU member states in the development of the upcoming legislative proposal on the EHDS for primary (EHDS1) and secondary use of health data (EHDS2). One of these was the launch of the Joint Action (JA) Towards the European Health Data Space (TEHDAS) in February 2021. The aim of TEHDAS is to support the European Commission (EC) and Member States (MS), identifying the challenges and providing concepts and options for the EHDS2. It is built on the needs and expectations of national and international stakeholders, citizens’ engagement and existing infrastructure. The major expected outcome of this JA is a sustainable roadmap towards the EHDS2 implementation promoting the cross-border sharing of health data for secondary use. The EHDS2 will benefit population health by facilitating the timely exchange of and access to health data, improving not only the care of patients but also facilitating research, epidemiology, disease prevention and data-based policy decisions. This workshop aims to provide an overview of the preparedness of MS to join the EHDS for secondary use of health data, covering current state-of-play of health data management systems in the EU, barriers on the secondary use of health data, the ongoing work on the development and deployment of the EHDS2 infrastructure and finally, how population health research can benefit from the EHDS2. The workshop will begin with a short introduction on the TEHDAS JA, followed by a presentation on the outcomes of the TEHDAS country visits. In these country visits, the state-of-play of the health data management system and the preparedness to join the EHDS2 were mapped in 12 different EU MS. The next presentation will continue with the results from a study that collected more evidence on the barriers to cross-border sharing of health data for secondary use and options on how to overcome these. It will focus on the legal preparedness and barriers and will discuss how the options proposed by experts in the field align with the legislative proposal for the EHDS2. The audience will be able to give their opinion on the different options through an interactive voting poll during the session. Some of the options on how to overcome technical barriers will be taken further in the development of a pilot infrastructure for the EHDS2. Therefore, the last two presentations will focus on the development and deployment of this pilot infrastructure for the EHDS2 and the role and benefits that population health research will have in it. Throughout the workshop there will be discussion sessions between presentations to allow exchange of knowledge, experiences and opinions with the audience.

**Key messages:**

• The main outputs will support EU countries to prepare their health data management system for the EHDS2 based on the best practices and actions presented.

• The presentations will inform and align participants on the current work on the technical EHDS infrastructure that is being developed for secondary use of health data, the EHDS2.

